# Biotransformation of steroidal saponins in sisal (*Agave sisalana* Perrine) to tigogenin by a newly isolated strain from a karst area of Guilin, China

**DOI:** 10.1080/13102818.2014.978199

**Published:** 2014-11-28

**Authors:** Yanchao Wang, Xia Li, Hao Sun, Kexian Yi, Jinlong Zheng, Jie Zhang, Zaibin Hao

**Affiliations:** ^a^ College of Chemistry and Bioengineering, Guilin University of Technology, Guilin, China; ^b^ College of Life Science, Northeast Agriculture University, Harbin, China; ^c^ Environment and Plant Protection Institute, Chinese Academy of Tropical Agricultural Sciences, Haikou, China; ^d^ State Key Laboratory for Biology of Plant Diseases and Insect Pests, Institute of Plant Protection, Chinese Academy of Agricultural Sciences, Beijing, China

**Keywords:** sisal, tigogenin, biotransformation, steroidal saponins, acid hydrolysis

## Abstract

A rod-shaped bacterium was isolated from the soil in a karst area of Guilin, China and its biotransformation of steroidal saponins in sisal (*Agave sisalana* Perrine) to tigogenin was presented for the first time. A total of 22 strains for the degradation of steroidal saponins in sisal were isolated from 48 soil samples, and the isolated rod-shaped, bacterial strain ZG-21 was used for the production of tigogenin due to its highest degradation efficiency of steroidal saponins in sisal. The parameters affecting biotransformation by strain ZG-21 were optimized. Under the optimized conditions of temperature (30 °C), pH (6), time (5 days) and substrate concentration (5 mg/mL), a maximum tigogenin yield of 26.7 mg/g was achieved. Compared with the conventional method of acid hydrolysis, the biotransformation method provided a clean and eco-friendly alternative for the production of tigogenin.

## Introduction

Sisal (*Agave sisalana* Perrine), a member of the Agavaceae family, is a monocotyledonous plant originally from Mexico.[1–3] Sisal is of great economic interest because it is considered as an important source of hard fibres in semi-arid regions.[1,4] However, only a small proportion of sisal leaves are used for the production of fibres.[4] In order to make full use of sisal, sisal waste has been used as organic fertilizer, animal feed as well as a raw material for medicine including steroidal saponins.[3–6] In addition, close attention has been paid to the sisal waste because of the presence of steroidal saponins,[7–9] which possess a variety of biological and pharmaceutical activities, such as anti-proliferation, anti-anoxia, anti-inflammation.[10–13] Steroidal sapogenins are obtained by hydrolysing steroidal saponins. As one of the most important steroidal sapogenins, tigogenin is an important pharmaceutical raw material for the synthesis of steroid drugs [14,15] and can be extracted from the sisal waste after the production of sisal fibres. It was reported that tigogenin not only exhibited anti-inflammatory activity,[16] but also would be helpful in treating diabetes mellitus,[17] and preventing the development of osteoporosis and the related disorders.[18]

The synthesis of tigogenin was previously reported by Mazur and Sondheimer [19]. Nowadays, tigogenin is usually prepared by hydrolysing steroidal saponins in plants. Since tigogenin presents in a combined, glucosidal form called saponins in sisal, saponins must be subjected to hydrolysis to obtain tigogenin. Nonetheless, this method results in undesirable byproducts, high cost, environmental problems and hazards to human health due to the use of large amounts of toxic organic solvents. In recent years, biotransformation using microorganisms, also called microbial transformation, has attracted increasing attention of researchers, because it has the advantages of low cost, high specificity, mild reaction conditions and good environmental compatibility. Biotransformation has been used for the treatment of environmental pollution [20] and the production of natural products.[21] Up to date, there are many reports about microbial transformation for the preparation of diosgenin,[22–25] which is also an important steroidal sapogenin in the pharmaceutical industry. Recently, Deng et al. [26] found some strains hydrolysing saponins from *Agave sisalana* for the preparation of tigogenin. However, to the best of our knowledge, no investigations on the production of tigogenin using biotransformation were reported.

In the present work, a rod-shaped bacterial strain ZG-21 for tigogenin production was isolated from soil samples collected from a karst area of Guilin, China, and its biotransformation of steroidal saponins to tigogenin was investigated in detail for the first time. The parameters influencing biotransformation and acid hydrolysis of saponins were studied. The biotransformation method provided a new, cleaner and more environmentally friendly approach in producing tigogenin.

## Materials and methods

### Materials and chemicals

**Figure 1.**
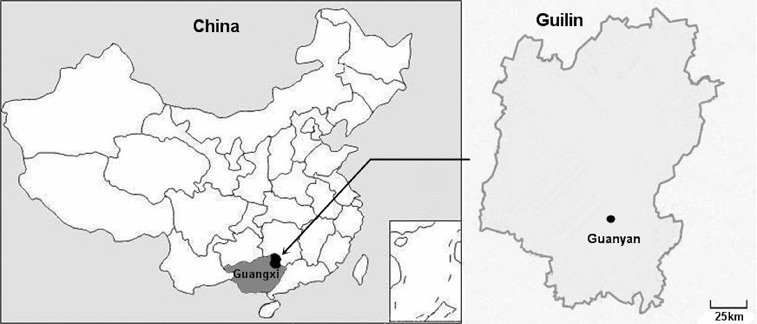
Sampling location of this study. Soil samples were collected from Guanyan, Yanshan, Guilin, Guangxi Province, China.

The sisal residue, after fibre extraction, was kindly provided by Guangxi Sisal Group Co. Ltd. (Guangxi, China). The raw material was dried and ground to a homogenous powder followed by being passed through a 100-mesh screen for further experiments. A total of 48 soil samples were collected from a karst area, Guanyan, Yanshan, Guilin, Guangxi, China (Figure 1), and used for isolation of target strains. All of the soil samples were collected at a depth of 3–5 cm after scrapping off the top layer and stored in sterile containers.

Methanol, HPLC grade, was obtained from Fisher Scientific Co. (Fair Lawn, NJ, USA). Tigogenin (97%) was purchased from Shanghai Tauto Biotech Co. Ltd. (Shanghai, China). Except otherwise stated, all other reagents were of analytical grade.[Fig f0002]

### Extraction of total saponins from sisal

Hundred grams of dried sisal residue powder was extracted with 100 mL of 60% ethanol using ultrasonic-assisted extraction for 1 h and the extract was filtered. The extraction was repeated two additional times with 50 mL of 60% ethanol for 0.5 h. Finally, the extracted ethanol solutions were combined and the ethanol was removed using a rotary evaporator (Model RE-52A, Shanghai Yarong Biochemical Instrument Factory, Shanghai, China) at 45 °C. The residue was successively extracted with 50 mL of petroleum ether, 50 mL of ethyl acetate and 50 mL of water-saturated n-butyl alcohol, respectively. The n-butyl alcohol was removed in a rotary evaporator at 55 °C. The residue was dissolved in ultrapure water to 50 mL. The saponin solution was further passed through a glass column (11 mm i.d. 500 mm length) packed with pretreated and preweighed hydrated D-101 macroreticular resin (equal to 1.0 g of dry resin) (Xi’an Sunresin New Materials Co. Ltd.) to remove interferences. Subsequently, the resin was eluted with 300 mL of ultrapure water, 300 mL of 30% ethanol and 500 mL of 80% ethanol, respectively. The eluate of 80% ethanol was collected and evaporated to nearly dryness in a rotary evaporator. The sample was finally dried to obtain powder by lyophilization.

### Isolation and screening of microorganisms from soil samples

The isolation of strains for degrading steroidal saponins was carried out according to the method of Zhang et al. [27] with some modifications. The screening medium was prepared by adding 2 g of the crude extract of total saponins from sisal, 2 g of agar powder, 0.001 g of ferrous sulphate, 0.05 g of magnesium sulphate, 0.05 g of dipotassium phosphate and 0.1 g of nitrate potash into 100 mL of water, while the preparation of the fermentation medium was as follows: the dried sisal residue powder was mixed with water at a weight/volume ratio of 100 g:1 L and the mixture was boiled for 2 h, the boiled solution was centrifuged at 4500 rpm for 15 min and the supernatant was diluted to 1 L with water and used as the fermentation medium. Both the media were sterilized at 121 °C for 20 min.

Two grams of each soil sample was suspended in 10 mL sterilized water. The mixture was shaken vigorously with a vortex mixer for 1 min and allowed to settle. Then 100 μL of the above supernatant was placed on the screening medium. The plates were incubated at 30 °C for 24–48 h. The isolates obtained from the primary screen were, respectively, placed into a flask containing 150 mL of fermentation medium and incubated at 30 °C in a rotary shaker incubator at 250 rpm. At the end of fermentation, the resulting fermentation broth was centrifuged at 4500 rpm for 15 min. The supernatant was dried and used for ultraviolet-visible (UV–visible) spectrophotometry analysis.

## *Morphological analysis*

A scanning electron microscope (SEM, JEOL, JSM-6380LV, Tokyo, Japan) was used to characterize the morphology of microorganisms. The fresh plate cultures were washed three times with physiological saline and then were fixed in 1% lysine solution. The above cultures were subsequently fixed with 2.5% glutaraldehyde at 4 °C for 2 h followed by being rinsed three times with physiological saline. The samples were further washed three times with physiological saline after being treated with 2% tannic acid for 2 h. The specimens were then serially dehydrated with a graded series of ethanol (30%, 50%, 70%, 90% and 100%) and air-dried, mounted on metal stubs. Finally, they were sputter-coated with gold for SEM analysis.

## *Molecular identification, physiological and biochemical tests of ZG-21 strain*

Genomic group DNA from strain ZG-21 was extracted using a DNA Extraction Kit (TIANGEN, China, centrifugal column form) according to the manufacturer's instructions. A bacterial universal primer pair (27F [5′-AGAGTTTGATCATGGCTCAG-3′] and 1492R[5′-TACGGTTACCTTGTTACGACTT-3′]) was successfully used for PCR (polymerase chain reaction) amplification of the 16S rRNA gene. The PCR product was detected using 1% agarose gel electrophoresis and sequenced by Sangon Biotech (Shanghai, China). The sequences were obtained and then analysed using BLAST analysis (http://www.ncbi.nlm.nih.gov/blast). Phylogenetic trees were constructed by the neighbour-joining method using MEGA 5.0 program after multiple alignments of the sequence data, Selecting *Bacillus sp*. DX-5 (KC311559) as a tree root.[28] The ZG-21 strain was cultured for 2 days at 30 °C in potato dextrose agar (PDA) medium. The strain was identified by colonial morphology, microscopic examination and biochemical characterization. There are some biochemical characterizations, such as the production of oxidase and catalase, lipid, starch and casein hydrolysis, gelatin liquefaction, nitrate reduction, and whether other carbohydrate as only carbon source can be available.

### Optimization of biotransformation conditions

We chose an isolate giving highest degradation efficiency of steroidal saponins for biotransformation of saponins to tigogenin. In order to reduce production cost and make the selected strain suitable for the actual production of tigogenin in industry, we used the supernatant of boiled sisal residue as the sole nutrient source of microorganisms. The parameters influencing the biotransformation were optimized in detail to obtain a higher yield of tigogenin. The effects of fermentation temperature, pH, time and substrate concentration on the yield of tigogenin were studied by changing the levels of the investigated parameter one-at-a-time while keeping the other three parameters constant. The resultant fermentation broth was centrifuged at 4500 rpm for 15 min. Then the precipitate was washed three times with ultrapure water and the supernatant was removed. Five millilitres of chloroform was utilized to extract tigogenin from the precipitate in combination with ultrasonic-assisted extraction for 30 min. The chloroform extract was used for purification of tigogenin.

### Optimization of acid hydrolysis conditions

In our work, acid concentration, hydrolysis temperature and time for the acid hydrolysis process were optimized in the same way, which was used for the optimization of biotransformation procedure described above. Hundred milligrams of dried sisal powder and HCl at different concentrations were added into a test tube. The mixture was heated in a water bath at different temperatures for different periods, and the tube was then cooled using cold water to terminate the reaction. Subsequently, the pH value of the cooled mixture was adjusted to neutral using 6 M NaOH. The solution was centrifuged at 4500 rpm for 15 min and the precipitate was extracted three times with 10 mL of petroleum ether (b.p. 60–90 °C). The petroleum ether extracts were combined followed by centrifugation at 4500 rpm for 15 min. Then the precipitate was washed with ultrapure water and the supernatant was removed. The following treatment was the same as that of fermentation broth mentioned in Section 2.5 and the resulting chloroform extract was also used for further purification of tigogenin.

### Purification of tigogenin

The products, which were obtained from biotransformation by the selected strain and acid hydrolysis, were further separated and purified with conventional silica gel column chromatography to give tigogenin fraction. The sample was loaded onto a glass column (11 mm i.d., 500 mm length) packed with silica gel (200–300 mesh, Qingdao Jiyida Silica Reagent Factory, Qingdao, China) using chloroform/methanol/water (80:20:5, v/v/v) as the mobile phase at a flow rate of 1 mL/min. The fractions were collected into test tubes at 2 mL/tube using an automatic fraction collector (model DBS-100, Shanghai Qingpu Huxi Instrument Factory, Shanghai, China). The tubes containing crystals were successively concentrated and lyophilized and then were analysed by HPLC-ELSD method.

### Analytical methods

The content of total saponins was detected with a UV–visible spectrophotometer using the method as described by Ma et al. [29]. A calibration curve with a correlation coefficient of 0.998 was prepared using standard tigogenin. The standard solution of tigogenin at a concentration of 1 mg/mL was prepared with methanol. Different volumes (0.2, 0.4, 0.6, 0.8 and 1.0 mL) of the standard solution were transferred to test tubes and the methanol was removed. After cooling, 0.2 mL of 5% (w/v) vanillin/glacial acetic acid solution and 0.8 mL of perchloric acid were, respectively, added into each tube. All of the above solutions were heated in a water bath at 70 °C for 15 min and then were immediately cooled using cold water. Finally, 5 mL of glacial acetic acid was placed into each tube and the absorbance was measured at 450 nm using a Cary 50 UV–visible spectrophotometer (Varian, Palo Alto, CA, USA). The content of total saponins was detected in the same manner.

The purified tigogenin was analysed using a high-performance liquid chromatography (HPLC) (model LC-20AB, Shimadzu Co. Kyoto, Japan) system equipped with an evaporative light-scattering detector (ELSD) (model ELSD-LT II Shimadzu Co. Kyoto, Japan). A Shim-pack VP-ODS C_18_ column (250 mm × 4.6 mm i.d. 5 μm particle size, Shimadzu Co. Kyoto, Japan) was utilized as the separation channel at a column temperature of 30 °C. The mobile phase for isocratic separation consisted of methanol/water (90:10, v/v) at a flow rate of 1.0 mL/min. An aliquot of 10 μL of each sample was injected into the HPLC system. The drift tube temperature was controlled at 40 °C. Nitrogen was used as the carrier gas at an inlet pressure of 350 kPa.

### Statistical analysis

All results were obtained from three repeated experiments. Standard deviation was determined using Microsoft Excel.

## Results and discussion

### Isolation and screening of microorganisms from soil samples

**Figure 2. f0002:**
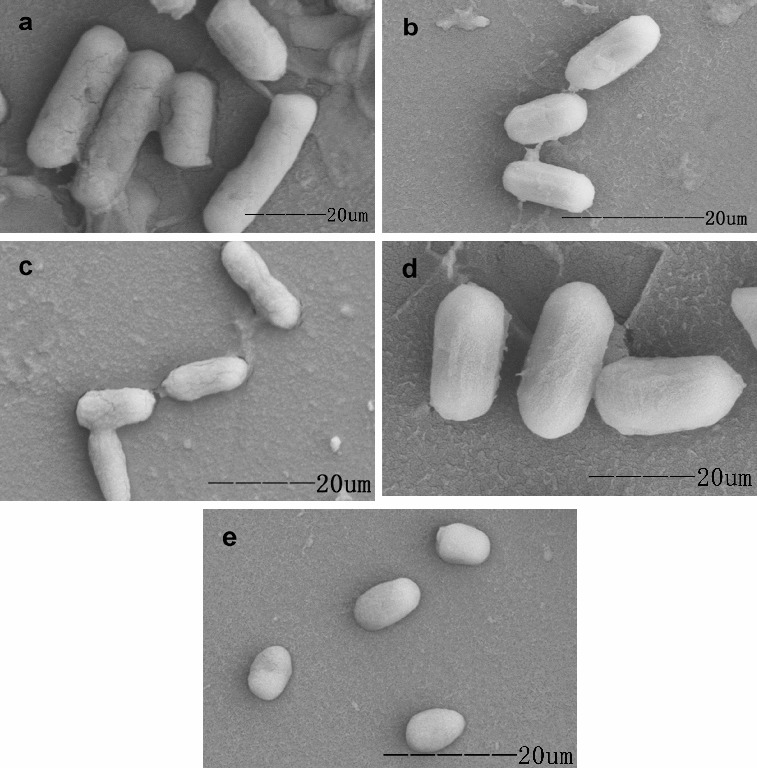
Morphology of some isolates from soil samples. (a) ZG-21; (b) ZG-04; (c) ZG-19; (d) ZG-14. (e) ZG-15.

The landscape of Guilin belongs to the karst topography in terms of its scale and uniqueness. Guanyan is located in Yanshan district in the south of Guilin, Guangxi, China. Prior to this study, we had isolated *Bacillus thuringiensis* (Bt) strains,[30] and strains for the degradation of chlorophyll from the soil samples in Guilin.[31] In the present work, a total of 22 strains (Table 1 and Figure 2) were isolated from 48 soil samples. Deng et al. [26] had isolated both fungi and bacteria for hydrolysing saponins from sisal, and fungi exhibited higher degradation efficiency. Furthermore, fungi were found to be suitable for the production of diosgenin from *Dioscorea zingiberensis* C. H. Wright.[24,32] However, no fungi for degrading steroid saponins were found in this study. Based on colony characteristics and SEM analysis, all the isolates were bacteria and classified into two groups: rod-shaped and coccus-shaped.

**Table 1.** Isolates for degradation of steroid saponins from sisal and their corresponding degradation rates.

Strain	Bacterial morphology	Saponins content before biotransformation (mg/mL)	Saponins content after biotransformation (mg/mL)	Degradation rate (%)	RSD (%)
Blank	–	5.00	4.93	1.4	0.7
ZG-01	Rod-shaped	5.00	4.18	16.5	1.2
ZG-02	Rod-shaped	5.00	4.04	19.3	1.9
ZG-03	Rod-shaped	5.00	4.10	18.1	1.0
ZG-04	Rod-shaped	5.00	3.35	33.0	1.4
ZG-05	Rod-shaped	5.00	3.88	22.4	1.1
ZG-06	Rod-shaped	5.00	4.14	17.3	1.8
ZG-07	Rod-shaped	5.00	4.40	12.1	1.6
ZG-08	Rod-shaped	5.00	4.48	10.4	2.0
ZG-09	Coccus-shaped	5.00	4.57	8.7	1.3
ZG-10	Coccus-shaped	5.00	4.71	5.9	1.1
ZG-11	Rod-shaped	5.00	4.27	14.6	2.1
ZG-12	Rod-shaped	5.00	4.34	13.3	1.5
ZG-13	Rod-shaped	5.00	4.41	11.8	1.2
ZG-14	Rod-shaped	5.00	4.00	20.1	1.9
ZG-15	Coccus-shaped	5.00	4.53	9.4	1.7
ZG-16	Rod-shaped	5.00	4.22	15.6	2.2
ZG-17	Rod-shaped	5.00	4.67	6.7	1.8
ZG-18	Rod-shaped	5.00	4.46	10.9	2.0
ZG-19	Rod-shaped	5.00	3.95	21.1	1.9
ZG-20	Coccus-shaped	5.00	4.64	7.2	1.7
ZG-21	Rod-shaped	5.00	2.59	48.2	1.5
ZG-22	Coccus-shaped	5.00	4.75	5.1	1.6

As shown in Table 1, the highest degradation rate (48.2%) of steroidal saponins was obtained using strain ZG-21 according to spectrophotometry analysis. Therefore, strain ZG-21 was chosen for further experiments. Strain ZG-21 is a gram-positive, non-motile, non-sporeforming, strictly aerobic, rod-shaped bacterium with an average width of 1.0 μm and an average length of 2.5 μm Figure 2(a). It belongs to the genus *Brevibacterium*. Moreover, strain ZG-21 is now preserved in China Center for Type Culture Collection (CCTCC, No. M2013322, Wuhan, Hubei, China).

**Figure 3.**
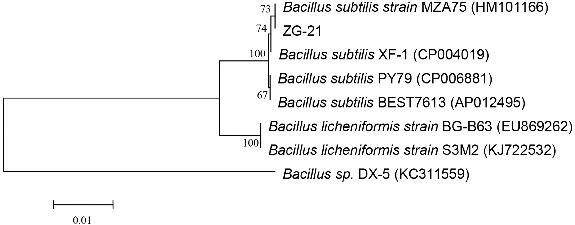
Neighbour-joining tree based on 16S rRNA gene sequences showing the phylogenetic position of strain ZG-21 and known bacteria from *Bacillus* sp. Numbers at nodes indicate the inferred values greater than 50% (1000 replicates), Bar 0.01 substitutions per nucleotide position.

The 16S rDNA fragment of ZG-21 was successfully amplified using designed primers, and a fragment of 1458 bp was obtained by sequencing. Phylogenetic trees were constructed by neighbour-joining method using MEGA 5.0 program after BLAST analysis (http://www.ncbi.nlm.nih.gov/blast) (Figure 3). Sequence analysis revealed that isolation strain ZG-21 showed above 99% homology with *Bacillus subtilis* strain MZA75(HM101166).[33] The biochemical characteristics of the isolate and carbohydrate utilization showed a positive reaction (Table 2), and the result was close to HM101166. Further identification results show that ZG-21 strain belongs to *Bacillus sp*.

**Table 2.** The physiological and biochemical characteristics of ZG-21 and *Bacillus subtilis* strain MZA-75 (HM101166).

Characteristics	ZG-21	HM101166
Colony morphology		
Shape	Round	Round
Size	Large	Large
Colour	White	White
Surface	Dull	Dull
Margin	Entire	Entire
Cell morphology	*Bacillus*	*Bacillus*
Gram staining	+	+
Spore formation	Central	Central
Biochemical tests		
Casein hydrolysis	+	+
Starch hydrolysis	+	+
Lipid hydrolysis	+	+
Gelatin liquefaction	+	+
Oxidase	+	+
Catalase	+	+
Nitrate reduction	+	+
Citrate utilization	+	+
Voges Proskauer	+	+
Methyl red	−	−
Urease test	−	−
Carbohydrate fermentation		
α-Cyclodextrin	−	
β-Cyclodextrin	−	
Glucose	+	+
Fructose	+	+
Sucrose	+	+
Mannose	+	+
Raffinose	+	+
Lactose	+	+
Sorbitol	+	+

Note: +, positive; –, negative.

### Optimization of biotransformation conditions for strain ZG-21

**Figure 4.**
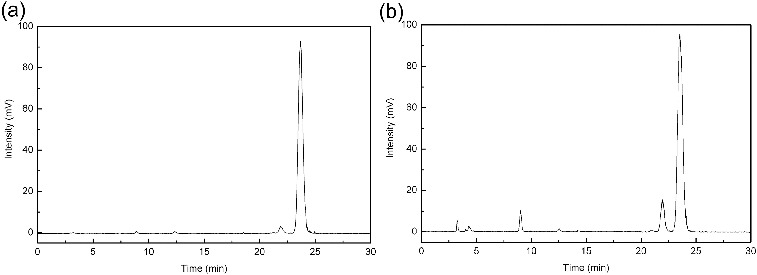
HPLC-ELSD chromatograms of standard tigogenin (a) and sample after biotransformation (b).

According to the above experiments, strain ZG-21 was used for the production of tigogenin due to its high degradation efficiency of saponins in sisal. To enhance the yield of tigogenin, fermentation conditions for strain ZG-21 were optimized by a single-factor-at-a-time strategy. A quantitative analysis of tigogenin was performed by the HPLC-ELSD method. A typical HPLC-ELSD chromatogram is shown in Figure 4. It illustrated that tigogenin was the main product after biotransformation. In our study, the raw sisal residue after boiling made up the fermentation medium. Fermentation temperature, time, pH value of the medium and concentration of substrate were investigated in detail, respectively.

**Figure 5.**
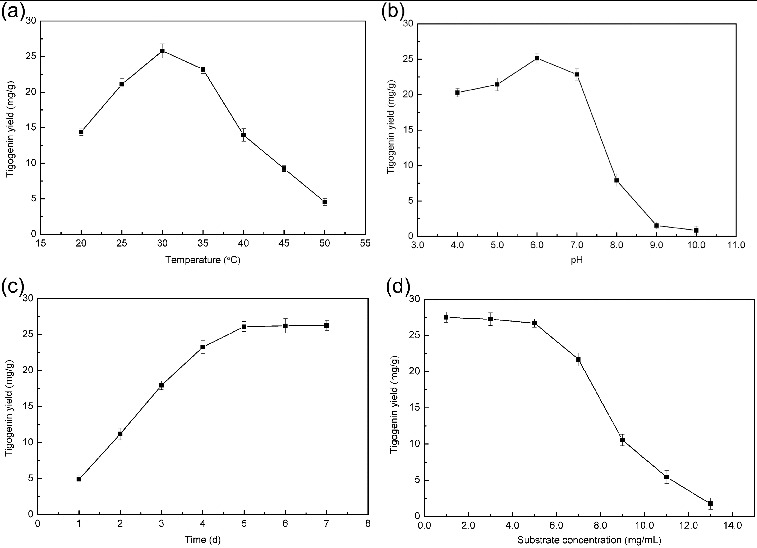
Effects of temperature (a), pH (b), time (c) and substrate concentration (d) on tigogenin yield. Conditions: (a) pH, 7.0; time, 4 d; substrate concentration, 5 mg/mL; (b) temperature, 30 °C; time, 4 d; substrate concentration, 5 mg/mL; (c) temperature, 30 °C; pH 6.0, substrate concentration, 5 mg/mL; (d) temperature 30 °C; pH 6.0; time, 5 d.

**Figure 6.**
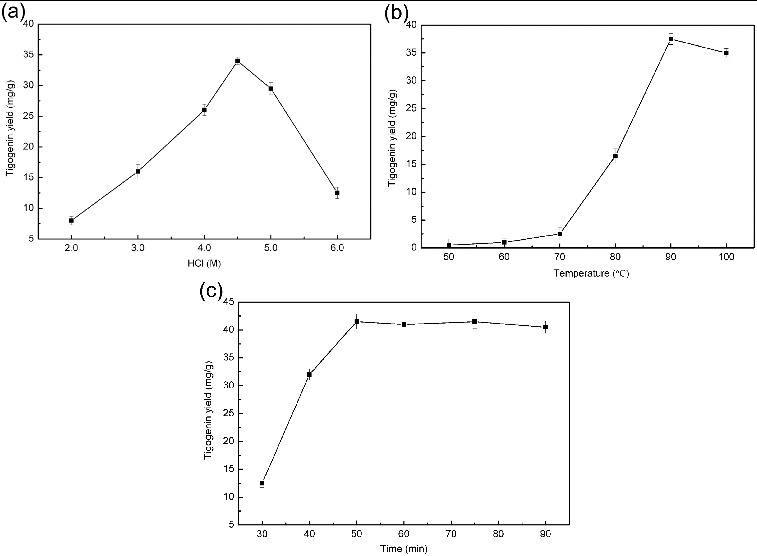
Effects of HCl concentration (a), hydrolysis temperature (b) and time (c) on tigogenin yield. Conditions: (a) hydrolysis temperature, 80 °C; hydrolysis time, 30 min; (b) HCl concentration, 4.5 M; hydrolysis time, 30 min; (c) HCl concentration, 4.5 M; hydrolysis temperature, 90 °C.

Temperature is an important factor for microbial growth and metabolism by influencing enzyme activity, substrate solubility, nutrient intake during the biotransformation process.[24,34] The effect of fermentation temperature on the yield of tigogenin was studied in the range of 20–50 °C. As shown in Figure 5(a), the yield of tigogenin increased when the temperature was varied from 20 to 30 °C, while the yield decreased when the temperature was higher than 30 °C. This was in accordance with previous works about biotransformation for producing diosgenin.[24,32] Therefore, the fermentation temperature was set at 30 °C for the subsequent study.

The pH of the medium also impacts on the production yield, substrate degradation, microbial growth and metabolism.[35,36] The effect of pH value on the yield of tigogenin was tested over the pH range of 4–10 (Figure 5(b)). It was found that the yield of tigogenin ascended in the pH range of 4–6 and then descended rapidly at higher pH values. The highest yield of tigogenin occurred at pH 6.0. Therefore, the medium at pH 6.0 was used for further experiments.

The influence of fermentation time on the tigogenin yield is displayed in Figure 5(c). The tigogenin yield kept increasing when the fermentation time was changed from 1 to 5 days. However, the yield tended to be stable after being incubated for more than 5 days. It may be because strain ZG-21 contributed to producing tigogenin at the beginning of incubation, but relevant enzymatic reactions in the production of tigogenin were inhibited with the accumulation of tigogenin and other metabolites. On the other hand, generally speaking, bacteria grow faster than fungi. As a result, the optimal fermentation time of strains for producing tigogenin was shorter than that of fugal strains for producing diosgenin.[32] Consequently, an incubation period of five days was adopted.

Substrate concentration can influence the substrate uptake and the efficient interfacial interaction between the enzyme and the substrate during the process and thus it affects tigogenin yield. The concentration of substrate was altered from 1 to 13 mg/mL to investigate its effect on the yield of tigogenin. As indicated in Figure 5(d), the tigogenin yield decreased with the increase of substrate concentration. Given that an obvious decrease occurred at concentrations above 5 mg/mL, the optimal concentration of substrate was fixed at 5 mg/mL. For the biotransformation process by strain ZG-21, the yield of tigogenin was 26.7 mg/g under the optimum conditions.

### Optimization of acid hydrolysis conditions

To better compared with the biotransformation method, the factors (acid concentration, hydrolysis temperature and time) influencing efficiency of acid hydrolysis for producing tigogenin were optimized to increase the yield of tigogenin.

The effect of HCl concentration ranging from 2 to 6 M on tigogenin yield was evaluated. As shown in Figure 6(a), the yield of tigogenin first increased and then decreased with the increasing HCl concentration. The highest yield was achieved using 4.5 M HCl.

The efficiency of hydrolysis for steroid saponins greatly depends on the temperature. The influence of temperature on tigogenin yield was investigated over the rage of 50–100 °C. As shown in Figure 6(b), the yield of tigogenin increased slowly when hydrolysis temperature was raised from 50 to 70 °C and then increased rapidly, but decreased above 90 °C. As a consequence, 90 °C was selected as the optimum hydrolysis temperature.

The investigation of the influence of hydrolysis time on tigogenin yield was carried out at different times ranging from 30 to 90 min (Figure 6(c)). As predicted, the yield of tigogenin first increased and then remained stable with the increase of hydrolysis time. According to Figure 6(c), no obvious increase in the yield of tigogenin was found when the hydrolysis time exceeded 50 min. Finally, a hydrolysis time of 50 min was selected for acid hydrolysis. For the acid hydrolysis process, the highest yield of tigogenin was 41.5 mg/g.

### Comparison of two methods

Under the optimized conditions, the yields of tigogenin obtained using biotransformation by strain ZG-21 and traditional acid hydrolysis were 26.7 mg/g, 41.5 mg/g, respectively. The yield achieved by transformation was 35.7% lower than that obtained by acid hydrolysis. The result indicated that the biotransformation approach suffers from the major drawback of low yield, which was similar to the previous work about diosgenin production.[37] Nevertheless, the biotransformation method can lead to less environmental problems with less acid and organic solvents. On the other hand, compared with the traditional acid hydrolysis process, Zhu et al. [24], Liu et al. [32] and Wei et al. [23] reported that a higher diosgenin yield by biotransformation could be achieved by optimizing various fermentation conditions. Therefore, further investigation needs to be carried out to provide higher tigogenin yield because of the complexity of enzymes during the biotransformation process.

## Conclusions

Biotransformation of steroidal saponins in sisal to tigogenin by a newly isolated strain was reported for the first time. A new rod-shaped bacterial strain ZG-21 was isolated from the soil in a karst area of Guilin and used for producing tigogenin. Under the optimal conditions, the yield of tigogenin via biotransformation was 26.7 mg/g, which was 35.7% lower than that of the traditional acid hydrolysis method. However, the new method had some advantages over the conventional method in offering low cost, less by-product and eco-friendly process. It provided an additional promising and valuable method for tigogenin production.
